# Voices of women in homelessness during the outbreak of the COVID-19 pandemic: a co-created qualitative study

**DOI:** 10.1186/s12905-023-02157-x

**Published:** 2023-01-10

**Authors:** Elisabet Mattsson, Marléne Lindblad, Åsa Kneck, Martin Salzmann-Eriksson, Anna Klarare, Women Advisory Board for Inclusion Health

**Affiliations:** 1 Department of Women’s and Children’s Health, Healthcare Sciences and e-Health, Uppsala University, 751 85 Uppsala, Sweden; 2 Department of Health Care Sciences, Marie Cederschiöld University, Stigbergsgatan 30, Box 11189, 100 61 Stockholm, Sweden; 3 Department of Health Sciences, Swedish Red Cross University, Box 1059, 141 21 Huddinge, Sweden; 4 Department of Caring Sciences, Faculty of Health an Occupational Studies, University of Gävle, 801 76 Gävle, Sweden; 5 Ersta Möjlighet, Stockholm, Sweden

**Keywords:** COVID-19 pandemic, Homelessness, Inclusion health, Public involvement, Qualitative, Women

## Abstract

**Background:**

Women in homelessness face extreme health- and social inequities. It could be postulated that during societal crises, they become even more vulnerable. Thus, the aim was to explore experiences related to the COVID-19 pandemic among women in homelessness.

**Methods:**

Ten interviews were conducted with women in homelessness, in Stockholm, Sweden, using researcher-driven photo elicitation. The data analysis was guided by the DEPICT model for collaborative data analysis and a qualitative content analysis was performed. A collaborative reference group of women with lived experience of homelessness contributed to the research process through designing the data collection, performing the data analysis, and providing feedback during report writing.

**Results:**

For women in homelessness, the COVID-19 pandemic was *adding insult to injury*, as it significantly affected everyday life and permeated most aspects of existence, leading to diminished interactions with others and reduced societal support. Thus, in an already dire situation, the virus amplified health- and social issues to another level. The women strived to find their balance on the *shifting sands* of guidelines and restrictions due to the pandemic. Adhering to the new social distancing rules and guidelines in line with the rest of society, was simply impossible when experiencing homelessness. However, for some women the pandemic was nothing but *a storm in a teacup*. The harsh reality continued irrespectively, living one day at a time and prioritizing provision for basic human needs.

**Conclusions:**

The COVID-19 pandemic and homelessness can be viewed as two intersecting crises. However, the women’s aggregated experiences were greater than the sum of experiencing homelessness and meeting the threat of the virus. Gender, exposure to violence, poverty, social isolation, and substance use were additional factors that further marginalized the women during the pandemic. To rebuild a better and more sustainable post-pandemic future for all, global commitment to ending homelessness is crucial. In addition, addressing social determinants of health must be the number one health intervention.

**Supplementary Information:**

The online version contains supplementary material available at 10.1186/s12905-023-02157-x.

## Background

Homelessness is increasing in almost all countries within the European Union [1], and contributes to social division and disparities that become particularly prominent in times of pandemics. The outbreak of severe acute respiratory syndrome coronavirus 2 (SARS-CoV-2) in 2019, emphasizes an urgent need for prioritizing equitable societies, as the coronavirus (COVID-19) escalates existing health- and social discrepancies [2]. Vulnerable groups in society have been disproportionately affected by COVID-19, including increased mortality rates [3, 4]. The latter underscores the importance for professionals in health- and social care services to understand inclusion health and consequences of social exclusion. This is imperative to ensure that marginalized and underserved people in our societies, such as individuals experiencing homelessness, can access and benefit from the needed services, also during pandemics.

Inclusion health is defined as a policy-, research- and service agenda focusing health efforts on people in extremely poor health due to marginalization, multi-morbidity, and poverty [5, 6]. People who experience homelessness frequently suffer from multiple health issues, including deteriorating mental and physical health, and substance use disorder [5, 7]. In addition, discrimination and stigma [8, 9], poverty [6], violence and complex trauma [10, 11] constitute overlapping risk factors for poor health among people experiencing homelessness. This contributes to huge health inequalities signified by extremely poor health outcomes, often much worse than the general population, including premature death [5, 7]. Furthermore, there are differences between men and women, that must be understood and appropriately addressed in clinical practice. For example, unhoused women with substance use problems are exposed to an alarming degree of male violence [11] and the effects of homelessness significantly impact generations to come [12]. An increased risk of substance use and psychiatric disorders has been concluded in offspring whose mother had a history of homelessness. The health- and social inequities that women experiencing homelessness face cannot be understood from isolated factors such as ethnicity, class, or gender. Intersectionality provides a theoretical lens to consider the interactions between social factors such as age, class, disability, ethnicity, gender, religion or sexual orientation as interwoven instead of existing separately [13]. The concept represents an approach of identifying power relations and their impact on those who struggle most in society. However, homelessness is still viewed as a male phenomenon, thus, research regarding all aspects of the lives of women experiencing homelessness is scarce, especially in Europe [14]. In the wake of the pandemic, the specific impact of COVID-19 on women has been highlighted by the United Nations (UN) in a policy brief [15]: “Across every sphere, from health to economy, security to social protection, the impacts of COVID-19 are exacerbated for women and girls simply by virtue of their sex.” (p. 2). Globally, women earn less, have insecure jobs and live closer to poverty compared to men. During the COVID-19 pandemic economic and social stress deepened, and coupled with restricted movement and social isolation measures, gender-based violence increased exponentially. At the same time, healthcare and social service resources gravely decreased. Hence, it could be postulated that women in homelessness are even more vulnerable during pandemics.

It has been suggested that involvement of people who have experienced social exclusion in the research process can bring attention to underexplored research areas [6]. Thus, enabling those with lived experience to shape research design, topics for consideration, and the data collection and analysis process, can lead to a better understanding of the experiences, needs, resources and challenges facing the group in question. This can lead to the design of more appropriate policy and services—potentially improving access and uptake of healthcare services amongst the populations in question. Since the 1990s, public involvement is increasingly a core activity in research funding calls and best practice research guidelines [16]. The National Institute for Health Research [NIHR] in the United Kingdom (UK), defines public involvement as “research being carried out *‘with’* or *‘by’* members of the public rather than *‘to’*, *‘about’* or *‘for’* them” [17]. The intent with the collaboration is an active partnership between patients, carers, and members of the public, e.g., people with lived experience of the phenomenon under study, and researchers to influence and shape research priorities. The aim is to break down boundaries, share experiences, and build understanding in combination with actual influence on decision-making processes. There are multiple empirically grounded recommendations for conducting public involvement in research, including assigning set roles to all parties involved in the research process, having regular communication between researchers and end-users, setting clear end-user expectations, and training participating end-users in research skills [18]. However, public involvement rarely extends to the meaningful involvement of groups outside mainstream society [16], such as women experiencing homelessness. Some identified barriers that limit marginalized groups’ input in research and policy making are lack of knowledge [16] and recruitment challenges, e.g., finding representatives with different features, such as diversity, capacity to consider different perspectives, and accountability [19].

The COVID-19 pandemic has changed the world in ways we are challenged to understand. Striving toward a fairer and healthier society, as we emerge from the pandemic, warrants a shift in public involvement in research practice to enable the reciprocal involvement of inclusion health target populations [2, 16]. Involving marginalized and underserved populations in identification, prioritization, and translation of research findings into policies and practice has an impact on the realization of health equity [16]. Taking women’s vulnerability and right to participation as key points of departure, the aim was to explore experiences related to the COVID-19 pandemic among women experiencing homelessness.

## Method

### Design

This study is part of a research program striving to promote inclusion health among women in homelessness, by developing and implementing interventions to address health inequities, in collaboration with women with lived experience of homelessness. As a means towards this end, we developed an advisory board of researchers and intended research end-users, since this is a key strategy to support participatory research projects [20]. The research program adheres to the National Institute for Health Research [NIHR] definition of public involvement in research [17].

The present study aligned with a constructionist, qualitative paradigm [21], acknowledging that individuals have unique social realities, and utilizing the DEPICT model [22] to guide a collaborative analysis process. The Guidance for Reporting Involvement of Patients and the Public, GRIPP2, guided proceedings [23], see Additional file 1. The checklist aims to improve the quality, transparency, and consistency of the international public involvement evidence base, to ensure that public involvement practice is based on the best evidence.

### The Women Advisory Board for inclusion health

A collaborative reference group of women with lived experiences of homelessness was founded in the Spring 2020, the Women Advisory Board for inclusion health. The aim of forming the board was threefold: to ensure and promote public involvement in the research program; to consult with researchers on equal ground in interpreting and presenting research findings; and to gain methodological trustworthiness by joint prioritization in relation to the research agenda. The women lived in sheltered housing that offers specialized, tailored long-term support for women who struggle with substance use disorder, as well as exposure to male violence and abuse. The shelter has eight places and meets legal requirements in line with the highest national standards of security and confidentiality in Sweden, e.g., secret address, alarms, and security doors. Women were informed about the project and invited to participate by staff. Those who expressed an interest in attending were invited to weekly two-hour workshop(s), with the first and last authors (E.M. and A.K.). New women at the shelter were invited to participate consecutively, whereas women who were discharged had to leave the Women Advisory Board due to security reasons, i.e., to protect the identities of new board members. Thus, the constellation of board members was changeable. Women were free to attend depending on their schedule, psychological well-being, treatments, and interest, etc. Board members were paid per hour (temporary employment at Marie Cederschiöld University, Stockholm, Sweden) according to guidelines for patient and public involvement [24].

In the present study, the Women Advisory Board contributed to the research process by designing the data collection, i.e., operationalization of the photo elicitation method, data analysis proceedings, and providing feedback during report writing.

### Photo elicitation method

**Fig. 1:**
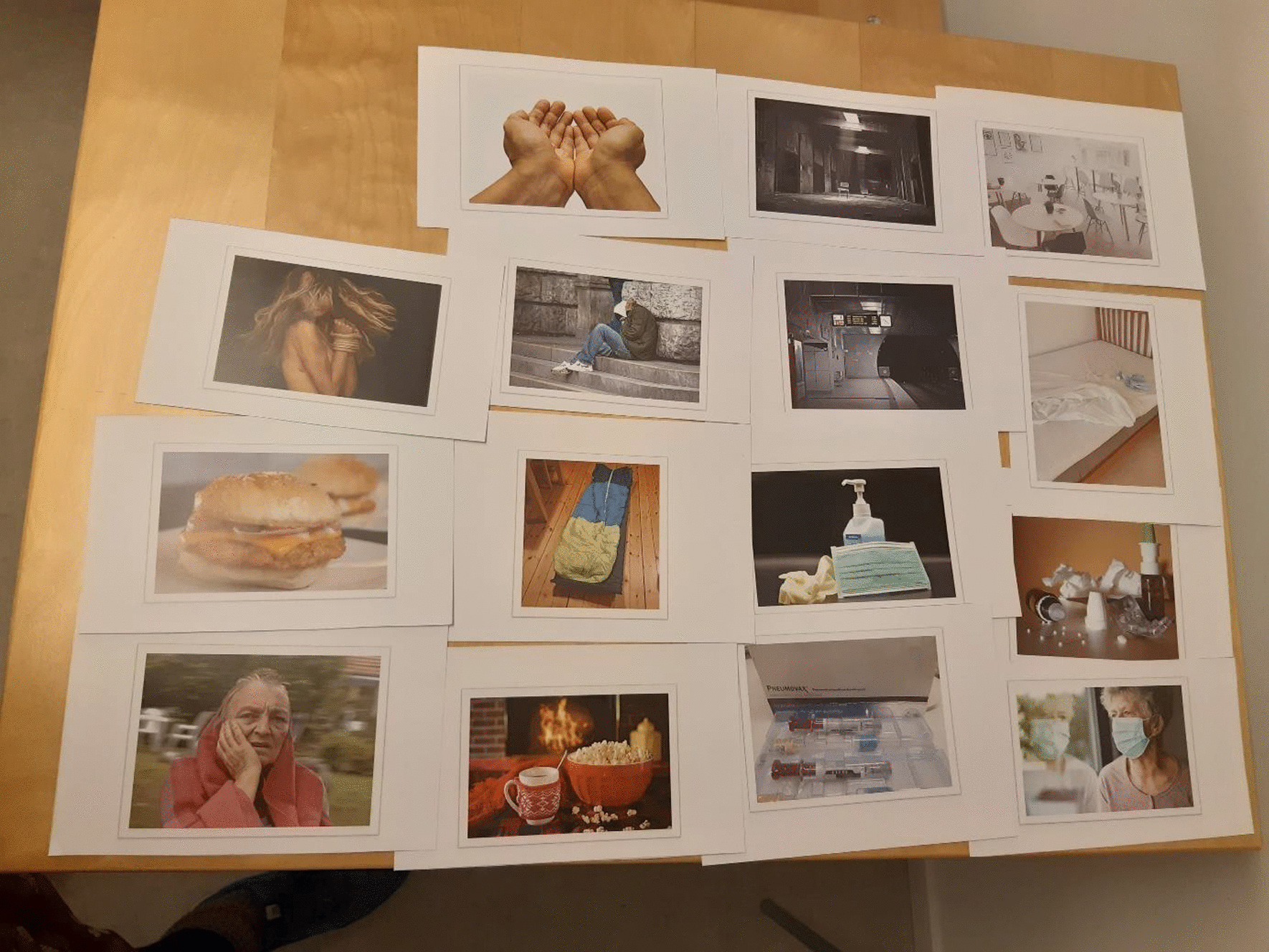
Photos (n = 15) selected by the Women Advisory Board for interviews to explore experiences of women in homelessness during the COVID-19 pandemic

During September 2020, three workshops (total 6 h) with the Women Advisory Board were conducted to outline the data collection procedures in this study. Three women and two of the authors (E.M. and A.K.) attended. The workshops included individual reflection, group discussions and brainstorming sessions focusing on engagement of women experiencing homelessness in active, meaning-making dialogues about their lives during the pandemic. Please see Additional file 2, for a description of content in each workshop. To summarize, we decided to use photo elicitation to increase participant-led dialogue in interviews [25]. Through a co-created process, 15 photos were selected by the Women Advisory Board (Fig. 1). Two photos were taken by the researchers (Å.K. and A.K.), whereas 13 photos, of which four included potential identifiable individuals, were downloaded from the website Pixabay.com. Pixabay is a vibrant community of creatives, sharing royalty free images, illustrations, videos, and music. Any content uploaded to the platform which features *identifiable* people must have an accompanying release from those identifiable people under which they give their consent for public usage of their images, please see the Pixabay License [Användarvillkor (pixabay.com)]. All content on the platform can be used freely for commercial and non-commercial use across print and digital media. The photos (n = 15) were subsequently used as stimuli to promote discussion during the interviews, i.e., a researcher-driven procedure [25].

### Setting

Sweden’s response to the COVID-19 pandemic was less invasive than many other countries, with no general lockdown [26]. During 2020, physical distancing was strongly recommended in public spaces, and mandatory in bars and restaurants. Face masks were initially not recommended outside healthcare, but eventually recommended for situations when maintaining distancing rules were problematic, such as in public transportation. Furthermore, no enforced quarantines for infected households or geographic regions were implemented. However, visits to nursing homes were banned to protect the frail elderly from the virus.

Sweden has large ethnic and socioeconomic inequalities in COVID-19 mortality [4]. Thus, the virus has been an additional burden on the most vulnerable individuals in society [3]. Practicing social distancing when living in unsheltered homelessness, including people living in abandoned buildings, cars, or parks, can be simply impossible. Universal strategies for prevention and control of infection in homeless shelters during the COVID-19 pandemic included bed spacing, limited staff rotation and reduction in the number of residents [27]. Thus, previously sheltered people had to move outside, as shelters shut down due to restrictions caused by the pandemic.

Homelessness among women is increasing in Europe [28, 29] and they constitute approximately 40 percent of the homeless population in Sweden [29]. However, it is important to bear in mind that the available statistics are unreliable. The true extent of women’s homelessness in Europe is, in fact, unknown and most likely underestimated [30].

### Data collection

Data collection took place in a primary healthcare center for individuals in homelessness in Stockholm, the capital of Sweden (Stockholm County, population 2.4 million). The center is open on weekdays and offers a broad array of healthcare services, without requiring pre-booked appointments. Visits are free of charge, and the center has close collaborations with social services, primary and psychiatric care, and services for treatment of substance use disorders. During 2020, the healthcare center had around 10,000 visits, and 23 percent were women.

Participants were recruited by a female researcher (third author, Å.K.) in the waiting area of the healthcare center, using convenience sampling. She had experience and training in using photos for research interviews. Potential participants were approached and informed about the study. Inclusion criteria were women with experiences of homelessness and speaking Swedish or English. Exclusion criteria were women exhibiting severe distress or anxiety, manifesting as violent or abusive behavior. If a woman wanted to participate, further written and verbal information were provided in an adjoining room, where the interviews were conducted.

At the start of the interview, the women faced a table with 15 photos comprising potential associations to life during the pandemic, see Fig. 1. They were invited to choose five photos that they associated with life during the ongoing pandemic, and to elaborate on their thoughts and feelings related to the photographs and/or the pandemic. Probes, such as “Could you please elaborate?” or “Would you tell me more about…?” were used to deepen the interviews. The interviews were audio recorded and lasted between 10 and 57 min, with a mean duration of 33 min. As a token of appreciation, the women received a gift certificate (20 euros) valid in a national chain of grocery stores.

### Participants

Between October to December 2020, interviews were conducted with 10 Swedish-speaking women with experiences of homelessness. The youngest woman interviewed was 31 years old and the oldest was 64 years old (median 46 years). Time in homelessness varied between three months to 25 years (median 9 years). However, three women could not specify how many years they had been in homelessness, but all stated it had been for the majority of their adult life. One of these women was the oldest at 64 years old.

According to the European Typology of Homelessness and Housing Exclusion (ETHOS) [31], most of the women (n = 7) were houseless, i.e., lived in homeless hostels, temporary accommodation, transitional supported accommodation, women’s shelter accommodation, reception centers or supported accommodation for formerly homeless persons. Three women were roofless, i.e., slept in public space or night shelters. Four women received financial aid from the social welfare, three had illness compensation and two women lacked financial resources. One woman worked in a state-funded company for people with disabilities.

When invited to choose photos, eight women selected between four to six photos, whereas two women chose not to select a photo. One stated that no photo matched how she experienced life during the pandemic, and the other woman was un-willing to select specific photos without giving a reason. All photos, except the one with a sleeping bag on the floor, were chosen. The most popular photos were chosen by five participants: an empty subway station and a photo illustrating a face mask, gloves, and disinfectant for hands, see Fig. 1.

### Data analysis

All interviews were transcribed verbatim by a professional transcriber and analyzed using qualitative content analysis, as described by Graneheim and Lundman [32] and elaborated on by Lindgren et al. [33]. The analysis process was iterative and conducted in close collaboration between the researchers and the Women Advisory Board. Eight weekly face-to-face workshops were held between April to June 2021, each comprising 2 h (total: 16 h). Five women attended three to eight workshops each (three workshops: n = 1; four workshops: n = 2; seven workshops: n = 1; and eight workshops: n = 1) and took part in the present data analysis. All women spoke Swedish, but two had other first languages. In addition**,** the first author (E.M.) and one woman from the Advisory Board spent 8 h together to outline the results and discussion in the present paper.

The first and last authors (E.M. and A.K.) facilitated the workshops and kept a password protected logbook available only to the authors of this manuscript in Microsoft Teams. The DEPICT model for collaborative data analysis [22] was used to guide and structure the process. The model includes six sequential steps: dynamic reading; engaged codebook development; participatory coding; inclusive reviewing and summarizing of categories; collaborative analyzing; and translating. However, in the present study five steps were used, i.e., participatory coding by individuals was excluded. See Table 1 for an overview of data analysis activities with the Women Advisory Board.

**Table 1:** DEPICT steps, the roles of the Women Advisory Board and guiding questions

Depict step	Member roles	Questions to answer
Dynamic reading	Individually review a subset of randomly chosen transcripts. Record notes on important concepts	What aspects related to women’s experiences of life during the pandemic seem to be crucial in these texts?
Engaged codebook development	Work together to list important ideas for categorizing data	Do we have the right categories? Do we all understand what codes mean and how to apply them? Do codes require further refinement?
Participatory coding^a^	–	–
Inclusive reviewing and summarizing of categories	Work together to develop category summaries	What are some key quotes?
Collaborative analyzing	Work together to depict main findings	What are our most important findings?
Translating	Develop a knowledge translation and exchange plan for sharing research results to all relevant stakeholders	How do we get the word out? Strategies and suggestions?

^a^This step was not performed, i.e., individual review and coding a subset of transcripts

**Fig. 2:**
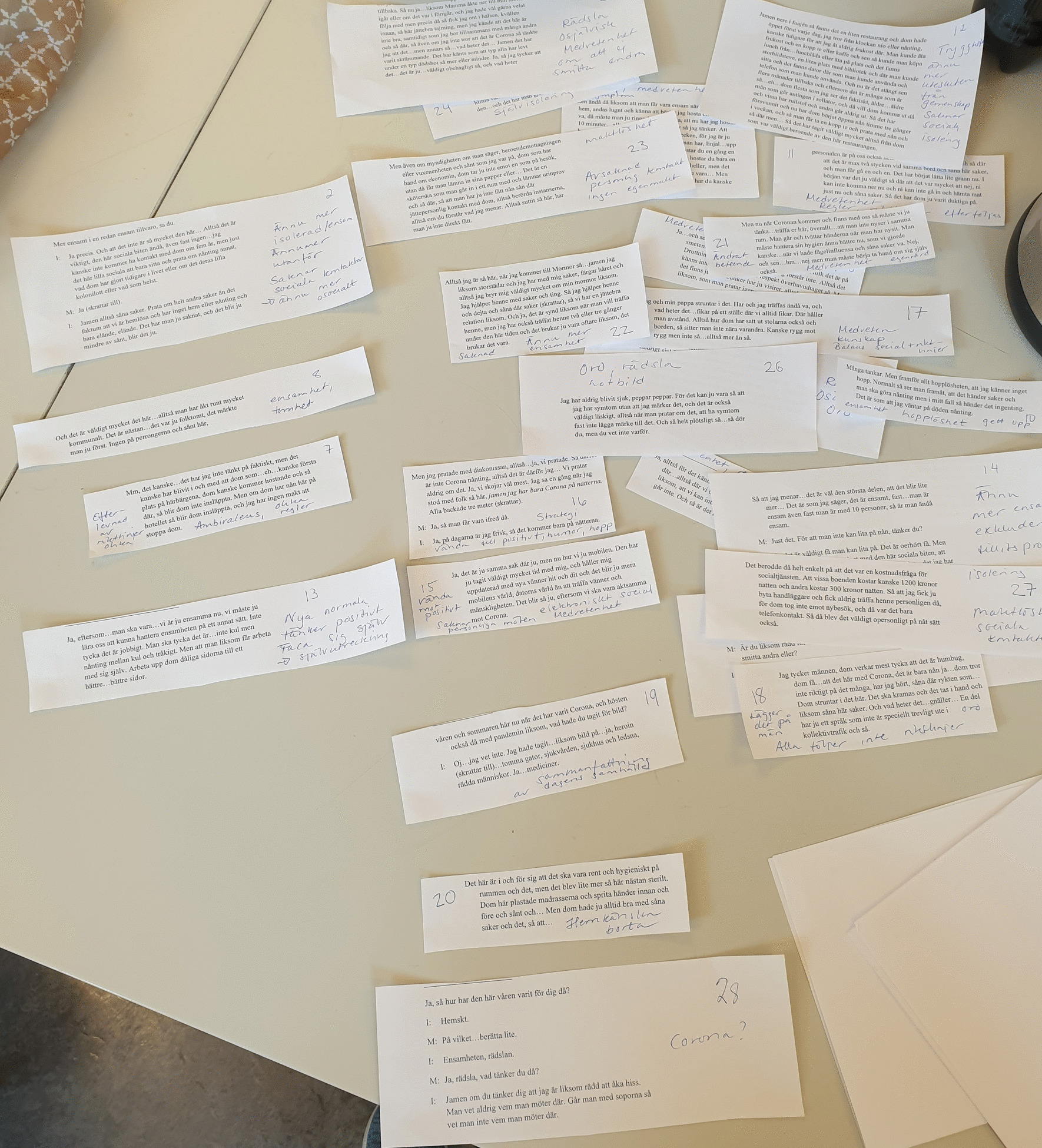
An example of analysis and grouping quotations

**Fig. 3:**
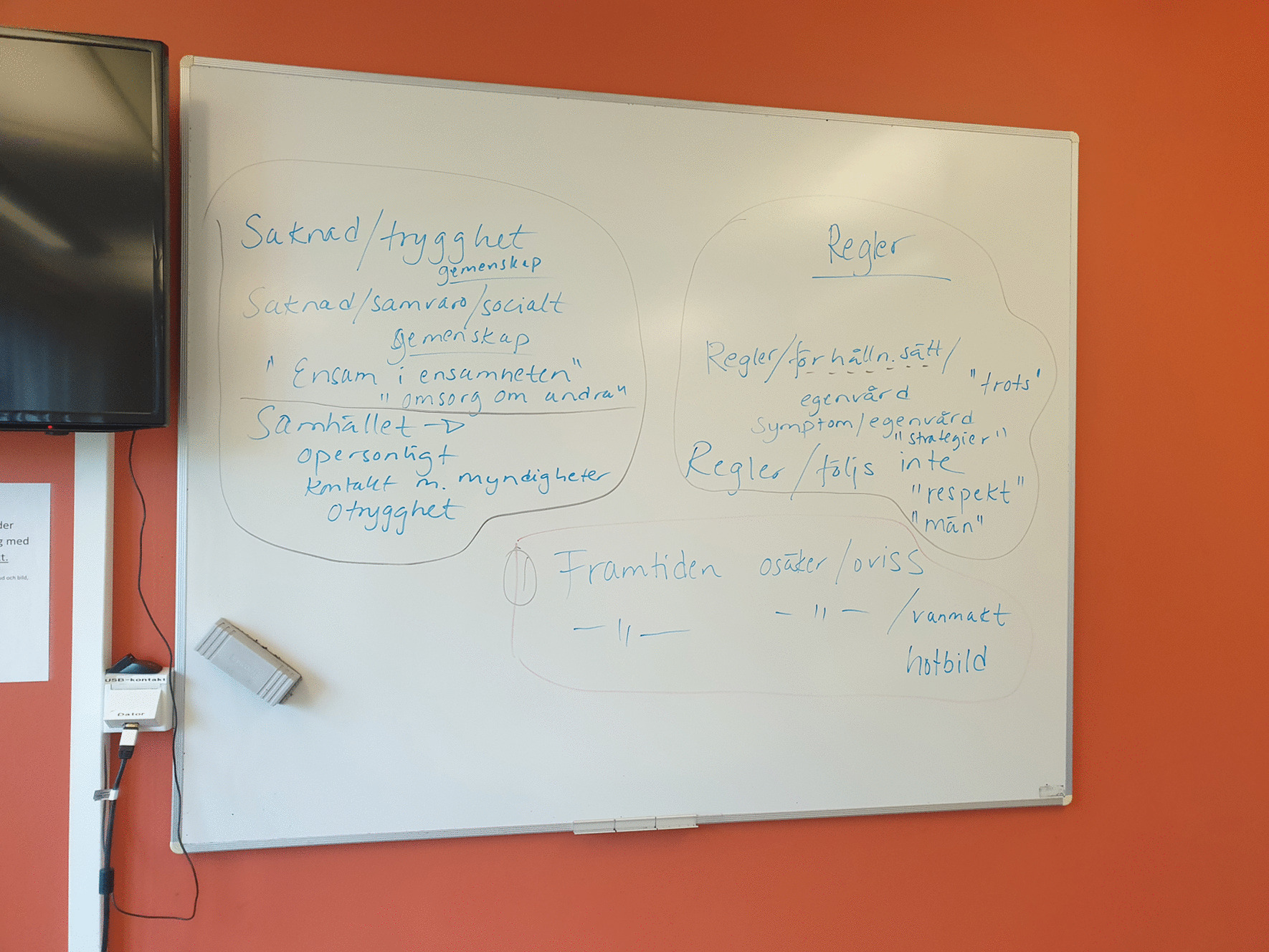
An example of the analysis procedure on the whiteboard

The first and last authors (E.M. and A.K.) read and re-read all de-identified interview transcripts (n = 10). One woman reviewed entire transcripts of randomly chosen (n = 2) interviews, whereas all members of the Women Advisory Board reviewed segments from all of the interviews. The last author (A.K.) used NVivo 12 for Windows to identify units of analysis, i.e., interview text relevant to the study aim. No automation tools or functions in NVivo were used. Identified units of analysis were written out on paper and presented to the Women Advisory Board. De-contextualization with codes and condensing was discussed [33], and general reflections were noted on a whiteboard, photographed, and documented in the logbook. Next, analysis continued with identifying meaning units, and labelling codes in collaboration with the Women Advisory Board. The codes were compared, highlighting similarities and differences, and subsequently grouped into categories with shared commonality. The categories were labeled and discussed in several workshops according to the same procedure as described above. These processes generated a first finding close to the interview narratives, i.e., manifest content [33]. Re-contextualization started with formulating sub-themes close to the aim, aiming to elucidate the unique experiences of women in homelessness during the COVID-19 pandemic. The Women Advisory Board identified main findings and chose key quotes related to the three sub-themes. To deepen the analysis and to abstract and interpret latent content, in collaboration with the Women Advisory Board, an overall theme was constructed aiming to make sense of the words in relation to the individual. The overall theme embraces the three sub-themes and highlights the underlying meaning within and beyond the text [33]. See Figs. 2 and 3 for illustrations of the analysis procedure.

The analysis procedure was conducted in several phases, including discussions and reflections among the authors and the Women Advisory Board, aiming to strengthen the trustworthiness and the credibility of the emerging findings [34]. In addition, to further deepen the latent content in the sub-themes [33], the first author (E.M.) and one woman from the Women Advisory Board reflected on the codes, categories, sub-themes and the overarching theme. This part of the analysis process was characterized by a forward and backward movement between the whole text and parts of the text. Reflections were summarized and subsequently discussed among the authors and the Women Advisory Board until consensus was reached.

### Ethical considerations

This study was performed in line with the principles of the Declaration of Helsinki. Ethical approval was granted by the Swedish Ethical Review Authority [2019-02130; 2020-02457].

All participants were offered written and verbal information about the study, and written informed consent was given by all participants. No identifying personal information that could be connected to a specific woman was collected. Demographics such as age, current housing status and occupation were documented. All data were self-reported, and no medical files were accessed. Audio recordings and all written documentation of the research data were stored on a password protected server, only accessible to the research group. Documentation was consecutively organized in folders, each folder contained the audio file, the transcribed text, the written consent form for that interview, and a photo of the chosen photos.

We were conscious of the fact that women who live/have lived in homelessness have difficult experiences of life, possibly characterized by abuse, trauma, and violence. Participating in interviews, as well as reading transcribed interviews, may trigger memories that cause significant distress for the interviewed women and the women attending Women Advisory Board workshops. Thus, attention and a concerted effort were focused on ensuring that the women had psychosocial support if needed in association with both the interviews and workshops.

## Results

The analysis resulted in an overarching theme: *The COVID-19 pandemic: yet another dimension of the world’s predictable unpredictability for women in homelessness*, represented by three sub-themes: *Adding insult to injury*, *Shifting sands,* and *A storm in a teacup*, see Table 2.

**Table 2:** Overview of theme, sub-themes and sub-themes content

The COVID-19 pandemic: yet another dimension of the world’s predictable unpredictability for women in homelessness	
Sub-theme	Sub-theme content
Adding insult to injury	Experiences that the ongoing pandemic significantly affected everyday life and permeated most aspects of existence, leading to diminishing interactions with others and reduced societal support. In an already lonely and unsafe life, the virus brought health- and social issues to another dimension
Shifting sands	Descriptions of adjustments and reorientation to a new reality dominated by guidelines and restrictions. For the women, the pandemic comprised yet another dimension of a fickle existence with sudden adjustments that they, due to homelessness, failed to incorporate in their lives, although they wanted and tried to
A storm in a teacup	Experiences that life was not changed because of the COVID-19 pandemic. The harsh reality continued irrespectively. The women described living one day at a time and prioritizing provision for basic human needs, e.g., finding food, a safe place to sleep, and escaping violence and abuse

Due to the COVID-19 pandemic women faced another dimension of the world’s predictable unpredictability. The women described that the on-going pandemic significantly affected everyday life and permeated most aspects of their existence. Living in homelessness comprises uncertainty and challenges to manage daily activities, these aspects were taken to another level during the pandemic. Adjustments and reorientation to a new reality dominated the women’s lives and they made efforts to be a small part of the pandemic society. However, there were also experiences that life was not changed because of the COVID-19 pandemic. The harsh reality continued irrespectively, with women living on the outskirts of society as per usual.

### Adding insult to injury

The first sub-theme pertains to experiences that the ongoing pandemic significantly affected everyday life and permeated most aspects of their existence, leading to diminishing interactions with others, diminished opportunities with reduced societal support, and further accentuated loneliness in an already lonely life. The impact of the pandemic was described on several levels by women in homelessness, as individuals, in interactions with peers, and with societal services.

According to the women, living in homelessness entailed having a lot of acquaintances but few friends. Alcohol and substance use were part of daily life, regardless of whether the women themselves used drugs or not. The importance of always being attentive, vigilant, and never trusting anyone was thus underscored. Yet, socializing with others in similar circumstances used to be an important part of life before the pandemic. With these opportunities being lost, a sense of connection in social communities was taken away. In line with adhering to social distancing guidelines, daily life and altered interactions accentuated experiences of loneliness even further.

“But it’s nice with the social bit, to be able to sit and talk. So that’s probably what I’ve noticed the most, that you’ve become even more lonely in loneliness, or whatever you call it. Peculiar.” (W1)

The importance of social connections and a chance to talk about other things, and another kind of life was highly valued, however, during the pandemic, this became much more challenging. Women described missing these types of interactions and personal contacts.

In addition, some of the services for people experiencing homelessness were shut down and closed until further notice. This was often done at short notice and without offering any alternatives. Several natural meeting points just disappeared, seemingly overnight, resulting in a sense of loss and disconnect. The women also described that it was almost impossible to sleep over at the homes of acquaintances, as they often had done before the pandemic. Viewed as potentially contagious, they were not welcome anywhere. The only option left was living on the streets, often 24/7. Several women raised concerns over an increase of older women sleeping rough because of the pandemic. Spending so much time on the streets fundamentally affected the women’s lives negatively.

The women elaborated on how dangerous rough sleeping was. To avoid being a victim of physical and/or sexual assault, the women needed to stay awake, sometimes for several days. This in turn, was not possible without using drugs, such as amphetamine. According to the women, heavy drugs such as heroin seemed to disappear from the streets during the pandemic. For women with severe substance use disorders, this was experienced as a critical problem as they feared forced abstinence. For these women, the outbreak of COVID-19 made a dire situation even worse.

“You noticed quite quickly that…, well, from March or April, it was all gone. And then we had to, at least in Stockholm, hmm… and then it turned up again, and got a little better after a month or so. And that month was like… Right, many of my friends started shooting instead because…, the ones who only smoked before started shooting up to get by, to avoid the worst withdrawal symptoms, and get it more concentrated.” (W3)

The opportunities for personal contact in relation to health- and social care services also changed due to the guidelines for social distancing. Previously, the women had been able to sit face-to-face to discuss circumstances and potential interventions, but these meetings were either cancelled or conducted over the telephone.

“…case management times get longer because it’s such long time. I’ve had to beg for food for… a year now, or half a year. Yeah, it’s rough.” (W8)

The loss of personal encounters was especially challenging when being assigned a new case worker.

“So, I had to change case workers and never got to see her in person, because they accepted no visitors, and then it was all done over the phone. It became very impersonal somehow.” (W1)

Having and managing to keep a mobile phone became a prerequisite to maintain contacts with health- and social care, as well as family and close friends, due to the pandemic. This in turn, added further to the women’s vulnerability as mobile phones were hard currency on the streets. The women described being robbed not only once, but several times. Worries of losing this lifeline, i.e., not being able to make telephone calls, as well as missed chances to establish personal connections and relationships, were described as heightening feelings of loneliness and isolation.

### Shifting sands

The second sub-theme pertains to adjustments and reorientation to a new reality dominated by guidelines and restrictions during the ongoing pandemic. For women experiencing homelessness, who constantly face the world’s predictable unpredictability, the pandemic entailed yet another dimension of unpredictability that they had to adjust to and incorporate into their lives.

The women strived to balance on the shifting sands of guidelines and restrictions due to the pandemic. They were busy making sense of, and adhering to, the new social distancing rules and guidelines in line with the rest of society. There were multiple examples of reflecting on and assuming responsibility for following guidelines by wearing a face mask, washing hands, and avoiding social contacts.

“… Sure, you think about the two meters, and you don’t see that many people. I’ve tried wearing a face mask, but it gets so warm. But I do disinfect my hands and… and I’ve done that much more now after corona.” (W7)

There were also descriptions of ongoing monitoring and screening of one’s own body, including thoughts about when the experienced symptoms might be COVID-19, and when they were not. Women who participated in needle exchange programs, appreciated being tested for COVID-19 every three months. An increased awareness of the importance of self-care and how to best protect oneself, and others, from the virus were described by the women.

“Now that corona comes and is with us, you have to think… that you don’t sneeze in the same room. You go and wash your hands after sneezing. You have to manage your hygiene even better now, maybe like during the bird flu and stuff. Hmm, and you have to start taking care of yourself too.” (W4)

However, the women knew they were striving for the unattainable, as it is not possible to self-isolate, or to fully follow sanitation guidance as a person in homelessness. Nevertheless, the women did their best to adapt, and to fit in and be a part of a society in flux. They were aware of public health official's urgent admonishing to stay at home and to avoid non-essential travel. However, none of that was of much use for women who spend their nights either sleeping rough or in shared homelessness accommodation. Instead, they faced new dimensions of the harsh reality since the lockdown of society meant fewer beds in the shelters. Consequently, the women were forced to spend hours per day on public transportation systems to find a safe haven for one night at time.

“And then the fact that one shouldn’t be out in public transportation and stuff too much, well, you know, social services, they send you back and forth and sort of… so it’s actually… I don’t think I’ve ever used as much subway, buses and trains as in the past six months.” (W1)

Lack of beds in the shelters also impinged on women who were fortunate to have temporary accommodation. These became overcrowded when persons in homelessness, who were less fortunate, tried to find shelter from the streets. This resulted in feelings of insecurity and powerlessness among the women who lived there.

“…they may come coughing and stuff, and then they’re not admitted [to the shelter]: But if they have someone here at the hotel, they get in, and I don’t have any power to stop them…// And then that you invite people in from the street, so there’s faeces and garbage. People can sleep a little…in.. the elevators over there, and you throw your garbage, conduct your toileting and smoke, I mean… in elevators and stuff. Sometimes I see [drug] trading too…” (W2)

For women in homelessness, deviations from guidelines and restrictions related to the pandemic were inevitable, due to several reasons; sometimes simply to ease accentuated feelings of loneliness caused by social distancing. One woman stated that she meets her father, pandemic or not.

“Me and my dad don’t care. Him and I meet anyway and what do you call it… have coffee in a place where we always have coffee. You keep your distance there. I mean, how they’ve place the chairs and tables, so that you’re not close to others. Maybe back to back but not… not more than that.” (W5)

In a life without the physical and psychological shelter of a home, the few bonds that still existed to family and close friends were very precious. In contrast to individuals with stable life situations, the interviewed women could not afford the "luxury" of opting out of relationships due to adherence to pandemic restrictions. Nevertheless, they were conscious of the stipulated guidelines and tried to do their very best to keep up with them. Thus, when guidelines were not followed by other people in society in general, they were surprised and upset as it, according to the women, signalized disrespect for others.

“…right smack in the center [of the city]. There are lots… do you know how many people there are on Queen Street, I mean still? I don’t get it. I mean, it feels like they have no respect whatsoever.” (W5)

The women tried coping with changes due to the pandemic, however, they were struggling with guidelines and restrictions created for insiders, or people integrated in society, not outsiders, like women experiencing homelessness.

### A storm in a teacup

The third sub-theme pertains to the contradictory experiences that life was not changed due to the COVID-19 pandemic. The harsh reality for the women continued irrespectively. The women described living one day at the time, prioritizing provision for basic human needs, e.g., finding food, a safe place to sleep and last, but not least, escaping violence and abuse. For the women, the pandemic was nothing but a storm in a teacup.

There were rumors on the street, and in various situations, of people who did not believe in the coronavirus nor in the pandemic. Life went on as usual and some did not care, or perhaps had not even heard about guidelines and restrictions due to the pandemic.

“I think the men, they seem to think this is all a hoax, the few… that this corona, it’s just some… they don’t believe in it. I’ve heard rumors that… They don’t care. They hug and shake hands and stuff like that.” (W1)

The struggle against the spread of the COVID-19 virus did not engage people in homelessness as they had their own battles to fight, the struggle for everyday survival.

For women experiencing homelessness and problematic substance use, the pandemic represented unnecessary concern about an unimportant matter. Having lived rough on the streets for many years, and managing to stay alive was, according to them, a sign that they were true survivors. Thus, the women described not being afraid of catching a virus. They had been through much worse battles.

“But we who live there [temporary accommodation], we’ve lived as usual, I mean there’s no difference. I mean, if you get it, you get in, but I think that we users do pretty ok since we’ve lived rough, we are pretty resistant.” (W6)

Women described they had other, more important, concerns and priorities in their daily lives than the virus. They underscored that their first and primary need was to obtain permanent housing. The longing for being able to close and lock a door of their own, to feel safe and secure was present in the women’s narratives. The one thing they wished for was getting out of their present life situation, including toxic relationships, that brought nothing other than misery.

“Yeah…, he has struck me so violently. I testified… it was probably … on his part last, it was two weeks ago now. I won’t do it again, let me tell you that, but I am a bit scared too…, well as long as he is locked up [I am safe]…” (W10)

Violence and abuse were never far away for the women. Escaping abusive situations had often been contributing factors to homelessness and living in homelessness entailed becoming victims of violence again. Hence, the women found themselves in a never-ending vicious cycle of violence, which in turn was far more relevant than the pandemic caused by the COVID-19 virus.

## Discussion

For women experiencing homelessness the outbreak of the COVID-19 pandemic represented a new dimension of the predictable unpredictability in society. The women had their own unique experiences of life during the pandemic, which we will discuss through the lens of inclusion health, intersectionality, and health literacy.

Based on our findings, COVID-19 and homelessness can be viewed as two intersecting crises. However, the women’s aggregated experiences were greater than the sum of homelessness and the virus. Gender, exposure to violence, poverty, social isolation, and substance use were additional factors that further marginalized the women during the pandemic. These factors can be labelled as social determinants of health, non-medical factors that influence health outcomes. The United Nations (UN) Sustainable Development Goals (SDGs) and the Agenda 2030, is a commitment to ensure a better and more sustainable future for all. *No one is left behind, by reaching the furthest behind first,* is a key feature in the discussions on the SDGs and represents a shift in thinking and subsequent confronting of global challenges [35]. In practice, however, the groups furthest behind were left even further behind during the COVID-19 pandemic [2, 3], as exemplified by the women in our study. From an intersectional perspective, our findings clearly show how women experiencing homelessness were affected profoundly harsher due to their living situation: a structural inequality. Meanwhile, the general societal discourse was that ‘we are all in this pandemic together’, indicating that restrictions, lock downs and social distancing applied to everyone. However, the *false* sense of solidarity has been raised in literature [36]. Thus, one central post-pandemic concern is how to ensure that the pandemic does not result in exacerbating health inequalities further in the future. In line with inclusion health [2], we argue that the pandemic has highlighted that we, more than ever, need commitment to social justice and health equity. Thus, targeting health- and social care policies, structures and management to underserved populations with multiple potentially stigmatizing circumstances is imperative [6], as they often face double disadvantages in health compared to both privileged and singly disadvantaged individuals [37]. The phenomenon is known as the *double disadvantage hypothesis* [38] and underscores the importance of attending to individuals’ lives, experiences, and well-being at unique intersections [37]. Nationally, regionally, and locally we need to address the causes of health inequalities [2, 5, 6], i.e., the social determinants of health. Only then can we reach the furthest behind first.

The causes of increased risk of COVID-19 infection and related hospitalizations and mortality among people experiencing homelessness [3] are an interaction between individual and structural factors [7, 39]. Individual factors include poverty, mental and/or physical illness, and substance use problems [7], whereas structural factors include high transmission rates in overcrowded homelessness shelters [39]. Furthermore, living in homelessness entails no options for isolation and quarantine [3]. The implementation of national lockdowns in many countries, to mitigate viral spread, accentuated an urgent need to house people in homelessness. Thus, it could be speculated that the COVID-19 pandemic made homelessness more visible. In England, for example, in March 2020 local councils were given 48 h and emergency funding to house rough sleepers from the beginning of the first lockdown in England [40]. England’s most at-risk homeless were rapidly moved to empty hotel rooms. Temporarily, the homelessness issues were solved within a matter of weeks. Evaluation of such initiatives reveals that hotel placement during the COVID-19 pandemic was associated with reduced acute healthcare use among people in homelessness with high use of multiple health services, compared to high users without a placement [41]. In Sweden, the government’s strategy focused on voluntary measures, with no general lockdown [26] and no emergency funding to re-house people in homelessness. Thus, the Swedish Public Health Agency’s advice to stay at home, to maintain social distancing, and to leave home only for essential purposes was meaningless for the women in our study. Impossible to adhere to, although they wanted to and tried to.

The pandemic was described as having a devastating impact on the women with substance use disorders. Changes to substance supply routes led to an unpredictable supply, which in turn led to compulsive use as well as risk for withdrawal, at a time with reduced healthcare capacity. There are examples from other countries, where authorities tried to decrease the reliance on illicit, and often toxic, substances. In Canada, an emergency, provisional safe supply program provided pharmaceutical-grade medications and beverage-grade alcohol in COVID-19 isolation hotel shelters for people with substance use disorders [42]. The program was associated with low rates of adverse events, i.e., overdose, and of high rates of successful completion of the mandatory 14-day isolation stay. However, it is important to bear in mind that we do not know much about women’s exposure to male violence and abuse at isolation hotel shelters. Some authors have emphasized the immediate risk of being trapped with their perpetrator who capitalized on the home confinement regulations [43, 44]. On the other hand, the women in our study described, that being forced to live on the streets around-the-clock meant increased risk of suffering from violence. Indeed, some women testified that they had to rely on various substances to stay awake and vigilant. They were afraid of being robbed and/or becoming exposed to violence and sexual abuse if they fell asleep outside. This is in line with the literature, concluding that gender inequalities and violence have been exacerbated by COVID-19, leading to diminished well-being among women [3, 15]. Pandemic or not, creating strategies to protect women from violence and abuse is imperative and aligns with fundamental human rights, that must be acknowledged and implemented worldwide.

Some women in our study described that life went on as usual, regardless of the COVID-19 pandemic. A possible explanation for this finding is that people experiencing homelessness neglect health needs, since priorities related to basic human needs, e.g., food, shelter, and security, take precedence [8]. However, another explanatory factor may be health literacy, defined by World Health Organization (WHO) as *the cognitive and social skills which determine the motivation and ability of individuals to gain access to, understand and use information in ways which promote and maintain good health* [45]*.* The pandemic has indeed shown that dissemination of information regarding important public health issues needs to be adapted to persons with low health literacy [46], which in turn often is found among people experiencing multiple, severe disadvantages [47]. Furthermore, the COVID-19 pandemic made us even more aware of the importance of digital health literacy, i.e., the ability to seek, find, understand, and appraise health information from electronic sources to address health problems. Our findings did not point to any specific needs of unmet digital health literacy per se, in terms of how to use technology. However, the women’s vulnerability was emphasized due to the imminent risk of being robbed for their mobile phones. Thus, the prerequisite for maintaining contact with others was a risk and may have posed a limitation for the women to access timely information. The pandemic accentuated an acute need for digital health interventions to provide people with access to vital health services, while minimizing their potential exposure to infection through social distancing [48]. To improve access to healthcare and social services, and to break social isolation, people experiencing homelessness in Great Britain received free mobile phones in an initiative recognizing that digital connectivity had become a key survival need in the wake of the coronavirus [49]. The program provided 2500 mobile phones to eligible recipients and was administered by a charity organization and a telecommunication company. The initiative is in line with a recent study, concluding that equipped with the basic tools of smartphone technology in combination with reliable public transportation, people in homelessness can engage in activities needed to self-manage their health and meet their social needs [50]. For the women who participated in our study, having access to a phone during the pandemic was regarded as a lifeline. However, mobile phones were a valuable commodity on the streets, something the women were aware of, as they had been repeatedly robbed of their phones. In contrast to Great Britain, no free phones were offered on a large scale to people experiencing homelessness in Sweden. A lesson learned from the COVID-19 pandemic is that rapidly implemented and scaled digital health innovations, may have had unintended consequences for health equity [48]. To reach the furthest behind first in the next inevitable pandemic, we must ensure that development of digital literacy is on par with basic literacy for multiple disadvantaged and underserved groups in society, in combination with access to quality digital healthcare [48]. Otherwise these groups will suffer from worse health outcomes compared to both privileged and singly disadvantaged individuals [38], as demonstrated during the COVID-19 pandemic [2, 3].

### Limitations

In line with inclusion health [6], a field which seeks to prevent and address health and social inequalities stemming from poverty, marginalization, multimorbidity, and social exclusion [2, 5, 6], this study was conducted in close collaboration with women having lived experience of homelessness. The Women Advisory Board contributed to the design of the study, the analysis, and drafting the manuscript. In an international perspective, such a research collaboration, that is, between researchers and women on the outskirts of society, is unique. Nevertheless, there are several caveats to consider.

During the COVID-19 pandemic, Sweden did not implement national lockdowns to mitigate viral spread [26]. This contrasts with most European countries, which in turn limits the study’s transferability. Another factor to consider when interpreting the findings, is that the interviews were conducted in the early phase of the pandemic before the vaccine against COVID-19 was available.

The sample constitutes of 10 Swedish-speaking women who all lived in homelessness according to a definition widely used in Europe [31]. The sample may be considered small, but women in homelessness are hard-to-reach [14].

We used researcher-driven photo elicitation [25] to collect data and fifteen photos were selected by the Women Advisory Board (Fig. 1). If the women had taken their own photos and brought them to the interview, we would have been able to more clearly attend to each woman’s individual life, experiences, and well-being at unique intersections [37]. Nevertheless, our impression is that the photos acted as “icebreakers” in interviews with women in homelessness, which also is in line with the literature [25].

The DEPICT model for collaborative data analysis [22] was a valuable tool to guide and structure the process. However, one step in the model was excluded, *participatory coding by individuals*, as two women had difficulties reading and writing in Swedish since they had other first languages. In addition, attention deficit challenges made it hard for the women to focus on long interview texts. Thus, one woman from the Women Advisory Board individually reviewed a subset of randomly chosen transcript, *dynamic reading* [22], whereas the other women read segments of all transcripts. The women’s struggles were also mirrored in the analysis; shifting from manifest content to interpreting latent content [33] was a time-consuming challenge for the women in the Advisory Board. Nonetheless, we argue that the collaborative analysis contributed to enhance the trustworthiness and the reliability of the study.

The women (n = 8) and the researchers (n = 2) who attended the Women Advisory Board workshops (n = 11) appreciated the teamwork, but the collaboration also included challenges. Continuity of members of the Women Advisory Board was impossible to accomplish. The reason for this was that we had to meet the shelter’s requirements of security and confidentiality. Three women contributed to the study’s design (three workshops), whereas five other women contributed to the analysis and drafting of the manuscript (eight workshops). To summarize, eight women with lived experience of homelessness contributed to different parts of the research process by working 30 h together with two researchers (E.M. and A.K.). We acknowledge and value each woman’s unique contribution to different parts of the research process and argue that the collaboration strengthens the study’s trustworthiness.

## Conclusions

We interviewed women in homelessness about their experiences during the COVID-19 pandemic. Based on our findings, we can conclude that the women’s aggregated experiences were greater than the sum of homelessness and the virus. Gender, exposure to violence, poverty, social isolation, and substance use were additional factors that further marginalized the women during the pandemic. There are numerous examples that showed that the women in our study were left behind, including reduced access to vital societal services and fewer beds in shelters which meant no other options except living on the streets. The latter was devastating for the women with increased risk of exposure to violence and sexual abuse. Our findings provide a sad picture of structural inequity when crises intersect for women, that is, COVID-19 and homelessness. Even though we were all in the crisis together, the way the pandemic affected women in homelessness was profoundly different compared to other more privileged groups.

To rebuild a better and more sustainable future for all after the pandemic, we need a global commitment to ending homelessness. In addition, addressing social determinants of health in close collaboration with inclusion health target populations must become the number one health intervention. This is imperative to ensure that people who are the most marginalized and underserved in society, the further behind, are reached first in the next, inevitable pandemic.

## Supplementary Information

**Additional file 1.** GRIPP2 short form.

**Additional file 2.** Data collection method – Workshop 1-3 with the Women Advisory Board.

## Data Availability

The datasets used and/or analyzed during the current study are available from the corresponding author on reasonable request.
