# Supplementary material for: Voices of women in homelessness during the outbreak of the COVID-19 pandemic: a co-created qualitative study
Source: BMC Womens Health. 2023 Jan 10;23:11. doi: 10.1186/s12905-023-02157-x (PMC9830620; doi:10.1186/s12905-023-02157-x)
Supplement: Supplementary file 1 — Additional file 1. GRIPP2 short form. [file 12905_2023_2157_MOESM1_ESM.docx]

# Additional file 1 | GRIPP2 short form

| **Section and topic** | **Item** | **Reported on page No** |
| --- | --- | --- |
| 1: Aim | Report the aim of PPI in the study | 6-7 |
| 2: Methods | Provide a clear description of the methods used for PPI in the study | 7-14; Additional file 2 |
| 3: Study results | Outcomes—Report the results of PPI in the study, including both positive and negative outcomes | The entire results section is formed and written as a result of the PPI |
| 4: Discussion and conclusions | Outcomes—Comment on the extent to which PPI influenced the study overall. Describe positive and negative effects | The women were part of the research group in data collection, analysis and report writing. We participated on equal ground, i.e. consensus decisions and striving for equal division of power. |
| 5: Reflections/critical perspective | Comment critically on the study, reflecting on the things that went well and those that did not, so others can learn from this experience | 30-31; Additional file 2 |

PPI=patient and public involvement
