# Supplementary material for: Voices of women in homelessness during the outbreak of the COVID-19 pandemic: a co-created qualitative study
Source: BMC Womens Health. 2023 Jan 10;23:11. doi: 10.1186/s12905-023-02157-x (PMC9830620; doi:10.1186/s12905-023-02157-x)
Supplement: Supplementary file 2 — Additional file 2. Data collection method – Workshop 1-3 with the Women Advisory Board. [file 12905_2023_2157_MOESM2_ESM.docx]

# ADDITIONAL FILE 2.

## Data collection method – Workshop 1-3 with the Women Advisory Board

In a previous interview study with women in homelessness (1), we used photo elicitation (2), a method of using photos as part of the interview process, to facilitate thinking out loud and reflection in interview situations. It has suggested that the method increases participant-led dialogue in interviews and therefore has the potential to produce rich data (2-4). Thus, we were interested in using the method for data collection in the present study.

## Workshop 1

The researchers (E.M. and A.K.) started the first workshop by describing different formats for photo elicitation (2), summarizing the literature and the research group’s experiences of using photos in interview situations with women in homelessness. The women attending the Women Advisory Board were positive to the method as they thought using photos may empower women in interview situations. The next step was to decide the format for the photo elicitation, i.e., participant-driven open (participants are asked to provide photos they feel relevant to the phenomenon of interest), participant-driven semi-structured (participants receive a set of questions and are asked to seek relevant photos to align with these) or research-driven (researcher provides the photos for the interview and uses these as stimuli to promote discussion) procedure (2). Potential pros and cons with each format were discussed and statements were written on a whiteboard. The women regarded participant-driven (open or semi-structured) formats to have the highest potential to introduce new dimensions about experiences of being a woman in homelessness during the COVID-19 pandemic. Dimensions that may not have been considered by the research group or the Women Advisory Board. However, due to the pandemic we decided that the photos were to be selected by the Board and would be used as stimuli to promote discussion during the interviews, i.e., a research-driven procedure (2).

## Workshop 2

The second workshop included a brainstorming session regarding the choice of photos. We applied the usual roles for brainstorming (5), i.e., women in the Board were encouraged to generate lots of suggestions and were told not to worry about their answers. There was no criticism or discussion regarding the legitimacy of suggestions which were generated during the workshop. The women were asked to suggest what they thought were significant situations in daily life related to the pandemic for women in homelessness. Suggestions were written on a whiteboard, discussed and subsequently, formulated into photo images e.g., an empty dining room at a shelter, a woman exposed to violence, masks, gloves, and disinfectants for hands, etc. After the workshop, another author (Å.K.) searched the Internet for free photos in accordance with the Board’s suggestions. Sixty photos were identified, of which 25 photos were selected by a research assistant with previous experience of homelessness (who was employed in the project). The photos were numbered and printed out before the third workshop.

## Workshop 3

During the third workshop, the Women Advisory Board was asked to choose photos that could be used in interviews with women in homelessness. The photos (n=25) were spread out in the conference room by the researchers (E.M. and A.K.) and the women received a pen and a paper and were encouraged to individually walk around and reflect on each photo, and if needed take notes. The following questions and prompts were used: Was the photo relevant to the study’s aim? Did it have the potential to promote dialogue in interview situations regarding how women in homelessness perceived their lives during the pandemic? Thereafter, the Board members shared their reflections, jointly discussed the photos, and grouped them into categories. These discussions related to drugs, empty spaces, home, pandemic-related utensils, portraits of women, and women in vulnerable situations. The Board unanimously selected 13 photos, however, they indicated that two photos were missing: a sloppy bed with materials indicating sex work, and a sleeping bag on the floor. After the workshop, two of the authors (Å.K. and A.K.) took photos in line with suggestions from the women. Thus, 15 photos were selected for the interviews, see Figure 1.

Insert Figure 1 about here

Figure 1. Photos (n=15) selected by the Women Advisory Board for interviews to explore experiences of women in homelessness during the COVID-19 pandemic.

## References

1. Kneck Å, Klarare A, Mattson E, Salzmann-Erikson M. Reflections on health among women in homelessness - a qualitative study. J Psychiatr Ment Health Nurs. 2022.  doi: 10.1111/jpm.12859.

2. Bates EA, McCann JJ, Kaye LK, Taylor JC. "Beyond words": a researcher's guide to using photo elicitation in psychology. Qual Res Psychol. 2017;14(4):459-81. doi: 10.1080/14780887.2017.1359352.

3. Meo AI. Picturing Students' Habitus: The Advantages and Limitations of Photo-Elicitation Interviewing in a Qualitative Study in the City of Buenos Aires. Int J Qual Meth. 2010;9(2):149-71. doi: 10.1177/16094691000900203.

4. Phipps M, Dalton L, Maxwell H, Cleary M. Combining Self-Determination Theory and Photo-Elicitation to Understand the Experiences of Homeless Women. Issues Ment Health Nurs. 2021;42(2):164-71. doi: 10.1080/01612840.2020.1789785.

5. Dunn WN. Public Policy Analysis: An Introduction: Pearson Prentice Hall; 2008.
